# Enhancing ribosomal translation of backbone-altering nonproteinogenic amino acids via YebC and YeeN

**DOI:** 10.1093/nar/gkag617

**Published:** 2026-06-22

**Authors:** Takayuki Katoh, Hiraku Takada

**Affiliations:** Department of Chemistry, Graduate School of Science, The University of Tokyo, Bunkyo-ku, Tokyo 113-0033, Japan; Department of Biotechnology, Faculty of Engineering, Toyama Prefectural University, Imizu-shi, Toyama 939-0398, Japan

## Abstract

Development of genetic code reprogramming methodologies has enabled the ribosomal incorporation of diverse backbone-altering nonproteinogenic amino acids (BAAs)—including d-α-amino acids, β-amino acids, and *N*-methyl-α-amino acids—into nascent peptides. However, their incorporation is far less efficient than that of canonical l-α-amino acids. We previously demonstrated that the ribosomal E-site-binding translation factors, EF-P and ABC-F proteins, can enhance BAA incorporation. These findings motivated us to investigate additional ribosome-binding factors that might further facilitate this process. Here, we focused on two putative A-site-binding factors, YebC and YeeN, to activate A-site BAA-tRNA and facilitate efficient incorporation. When testing 11 BAAs for consecutive incorporation, YebC and YeeN yielded average enhancements of 2.1- and 2.7-fold, respectively, with an increase of up to 6.6-fold in specific cases. Furthermore, combining YebC with EF-P and Uup (a representative ABC-F protein) produced an 8.0-fold increase in the incorporation of two consecutive *N*-methyl-l-leucine residues, demonstrating a clear synergistic benefit. We also found that YebC and YeeN promote ribosomal synthesis of drug-like macrocyclic peptides enriched with BAAs, such as d-serine, 1-aminocyclobutane-1-carboxylic acid, *N*-methyl-l-alanine, and β^3^-homomethionine. These results pave the way for the ribosomal synthesis of diverse macrocyclic peptide libraries and their application in mRNA display-based screening for novel bioactive compounds.

## Graphical Abstract

**Graphical Abstract:**
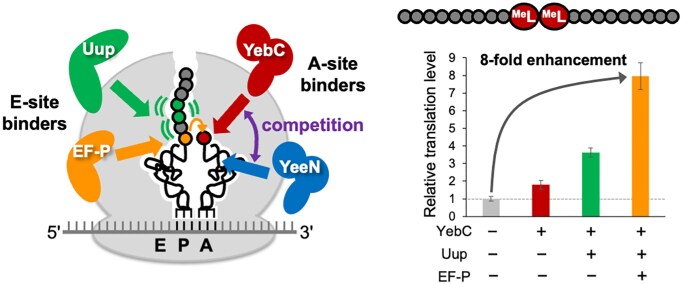

## Introduction

Backbone-altering nonproteinogenic amino acids (BAAs)—including d-α-amino acids, α,α-disubstituted α-amino acids, β-amino acids, γ-amino acids, and *N*-methyl-α-amino acids—are attractive building blocks for designing bioactive peptides. These BAAs introduce unique hydrogen-bonding patterns, steric effects, and conformational constraints distinct from those of canonical l-α-amino acids, leading to peptides with more rigid and well-defined structures [1–9]. Such foldamer-like peptides containing BAAs often display enhanced target-binding affinity, inhibitory potency, proteolytic stability, and membrane permeability [7, 9–21]. mRNA display-based discovery platforms, such as the random nonstandard peptide integrated discovery (RaPID) system, have enabled the identification of bioactive peptides containing such BAAs [20, 22]. Consequently, methodologies for the ribosomal incorporation of BAAs into random peptide libraries—amenable to mRNA display—have attracted growing attention. To date, various research groups have reported the incorporation of BAAs using *in vitro* translation systems [23–26]. In particular, our group has developed an *Escherichia coli*-based reconstituted *in vitro* translation platform, termed the flexible *in vitro* translation (FIT) system, which allows the ribosomal incorporation of a broad repertoire of BAAs [20, 27]. In addition to these cell-free approaches, cell-based systems utilizing orthogonal tRNA-aminoacyl-tRNA synthetase pairs have been developed by some groups to expand the genetic code with BAAs [28, 29].

However, the ribosomal translation of BAAs is often substantially less efficient than that of canonical l-α-amino acids [23, 30–33]. Multiple or consecutive incorporations are particularly challenging, hindering the generation of random peptide libraries containing several BAAs with quality sufficient for display-based screening. These inefficiencies primarily arise from three bottlenecks: (1) slow accommodation of BAA-tRNA at the ribosomal A site [34–38]; (2) slow peptide bond formation between A-site BAA-tRNA and P-site peptidyl-BAA-tRNA [30, 32, 35, 39]; and (3) ribosomal stalling induced by interactions between the BAA-containing nascent peptide and the ribosomal exit tunnel [40, 41]. We previously addressed issues (1) and (2) by developing an engineered tRNA, termed tRNA^Pro1E2^, which features specific T-stem and D-arm motifs that effectively recruit EF-Tu and EF-P to promote accommodation and peptide bond formation, respectively [39]. The weakened EF-Tu binding of BAA-tRNA can be compensated for by the enhanced EF-Tu affinity conferred by the T-stem motif, thereby facilitating ribosomal accommodation. EF-P, an E-site binding translation factor, naturally recognizes the D-arm of P-site peptidyl-Pro-tRNA^Pro^ to promote Pro incorporation [42–44]. Since tRNA^Pro1E2^ carries the same D-arm motif as native tRNA^Pro1^, EF-P can similarly recognize this tRNA and facilitate peptide bond formation of BAAs charged onto it. We also recently demonstrated that issue (3) can be alleviated by ATP-binding cassette family-F (ABC-F) proteins—the *E. coli* factors EttA, Uup, YbiT, and YhsS [41]. Structural and biochemical studies of diverse bacterial ABC-F proteins, including antibiotic resistance factors from Gram-positive bacteria, have shown that members of this family act as E-site binding ATPases. They remodel the peptidyl transferase center (PTase center) and P-site peptidyl-tRNA by inserting their interdomain linker, located between two ATP-binding domains, into the PTase, thereby rescuing stalled ribosomes [45–50].

The efficacy of E-site-binding factors like EF-P and ABC-F proteins suggests that they enhance the reactivity of P-site peptidyl-BAA-tRNA by optimizing its positioning within the PTase center. These observations motivated us to explore potential A-site-binding translation factors that might activate A-site BAA-tRNA and thereby promote its ribosomal incorporation. Recent studies reported that *E. coli* YebC family proteins, YebC and YeeN, facilitate polyproline peptide synthesis in a reconstituted *in vitro* translation system [51]. Similarly, *Bacillus subtilis* YebC2 (also known as YeeI) alleviates ribosomal stalling at polyproline sequences [52], while the mitochondrial homolog TACO1 in humans and mice is required for efficient polyproline translation [53]. Ignatov *et al*. investigated *Streptococcus pyogenes* YebC–23S rRNA interactions using iCLIP and identified cross-linked sites near helix 89—adjacent to the ribosomal A site—and nucleotides A2453, A2454, and A2456 (homologous to *E. coli* A2450, A2451, and A2453), all located within the PTase center [51]. These findings suggest that A-site Pro-tRNA becomes more reactive in the presence of YebC, resulting in enhanced Pro incorporation, although the precise mechanism remains unclear. Based on this evidence, we hypothesized that YebC family proteins could similarly promote the incorporation of BAAs by increasing A-site reactivity. In this study, we evaluated the translation-enhancing effects of YebC and YeeN on the incorporation of d-α-amino acids, α,α-disubstituted α-amino acids, β-amino acids, and *N*-methyl-α-amino acids. Furthermore, we assessed homologs from *Alteromonas macleodii* in combination with the *A. macleodii* ribosome—particularly for d-α-amino acids, d-β-amino acids, α,α-disubstituted α-amino acids, and *N*-methyl-d-α-amino acids—leveraging its inherently higher translational activity for these substrates compared to the *E. coli* ribosome [54].

## Materials and methods

### Preparation of mRNAs, tRNAs, and flexizymes

Template DNAs encoding mRNAs, tRNAs, and flexizymes were generated by primer extension and PCR using forward and reverse primer pairs (see Supplementary Table S1 for primer sequences). Extension and PCR were performed for 5 and 14 cycles, respectively, under the following conditions: 40 s at 94°C, 40 s at 50°C, and 40 s at 72°C. The resulting PCR products were purified by phenol/chloroform extraction followed by ethanol precipitation. Each template DNA contained a T7 promoter at the 5′ end, followed by the sequence corresponding to the mRNA, tRNA, or flexizyme. Template DNAs encoding mRNAs were used directly for transcription/translation-coupled reactions in the FIT system. Transcription of tRNAs was conducted at 37°C for 16 h in a reaction mixture containing 40 mM Tris-HCl (pH 8.0), 3.75 mM NTP mix, 5 mM guanosine monophosphate (GMP), 22.5 mM MgCl_2_, 1 mM dithiothreitol, 1 mM spermidine, 0.01% Triton X-100, 0.04 U/µL RNasin RNase inhibitor (Promega), 0.12 µM T7 RNA polymerase, and the corresponding DNA template. For flexizyme transcription, the NTP concentration was increased to 5 mM, and GMP was omitted. Transcribed RNAs were treated with RQ1 DNase (Promega) for 30 min at 37°C, recovered by isopropanol precipitation, and purified by denaturing polyacrylamide gel electrophoresis—8% for tRNAs and 12% for flexizymes—containing 6 M urea.

### Aminoacylation of tRNAs using flexizymes

BAAs, l-Ala, and l-Pro were preactivated as 3,5-dinitrobenzyl ester (DBE) or cyanomethyl ester (CME) derivatives as previously described [31]. *N*-chloroacetyl-l-phenylalanine (^ClAc^l-Phe) was prepared as a CME derivative, while all other amino acids were activated as DBE forms. These preactivated amino acids were then charged onto tRNAs using the appropriate flexizymes—aminoacylation ribozymes (dFx for DBE-activated substrates and eFx for CME-activated ones). Flexizyme reactions were performed at 0°C for 2, 6, or 24 h in mixtures containing 50 mM HEPES-KOH (pH 7.5) or Bicine-KOH (pH 9.0), 200 mM MgCl_2_, 20% DMSO, 25 µM dFx or eFx, 25 µM tRNA, and 5 mM activated amino acid. When using eFx, the MgCl_2_ concentration was increased to 600 mM. Reaction times, pH conditions, and flexizyme–substrate combinations for each amino acid are summarized in Supplementary Table S2. The resulting aminoacyl-tRNAs were recovered by ethanol precipitation, washed with 70% ethanol, and dissolved in 1 mM sodium acetate (pH 5.2) to yield 500 µM aminoacyl-tRNA solutions.

### Translation of model peptides using the FIT system

A reconstituted *in vitro* translation system, known as the FIT system, was used to synthesize model peptides containing BAAs [20, 27]. The specific compositions and reaction conditions for each experiment are detailed in Supplementary Table S3. Components of the FIT system, including translation factors, enzymes, and ribosomes, were expressed, purified, and mixed as previously described [27, 41, 42, 54]. Briefly, recombinant translation factors and enzymes were overexpressed in *E. coli* with hexahistidine tags and purified by affinity chromatography according to standard methodologies [55, 56]. Specifically, for the preparation of YebC and YeeN, genes from *E. coli* and *A. macleodii* were cloned into a pET28a-TEV vector. The plasmids were introduced into *E. coli* BL21(DE3) cells, which were cultured in LB medium and induced with 0.5 mM IPTG for 3 h at 37°C. Cells were lysed by sonication, and the lysate was applied to a His-TALON crude (5 mL) column (Cytiva) for affinity purification. The column was washed with buffer A [20 mM Tris-HCl (pH 8.0), 200 mM NaCl, 1 mM dithiothreitol] containing 10 mM imidazole, and His-tagged YebC or YeeN was eluted using buffer A with imidazole concentrations up to 300 mM. The eluted proteins were buffer-exchanged into imidazole-free buffer A and concentrated using Amicon Ultra centrifugal filters (Merck Millipore).

For the preparation of the *A. macleodii* ribosome, we followed a recently reported protocol [54]. *A. macleodii* ATCC 27126 was cultured in LBN medium supplemented with 10 mM MgSO_4_ until the mid-log phase. Harvested cells were disrupted by snap-freezing in liquid nitrogen followed by pulverization using a Multi-Beads Shocker (Yasui Kikai). The clarified lysate was subjected to hydrophobic interaction chromatography using Butyl Sepharose Fast Flow resin (Cytiva) with a decreasing ammonium sulfate gradient (1.5 to 0 M). Ribosome-containing fractions were pooled, layered onto a 30% sucrose cushion, and recovered by ultracentrifugation (100 000 × g, 16 h). The resulting pellets were resuspended in HEPES-Polymix buffer and stored at − 80°C.

### Analysis of translated peptides

For MALDI-TOF mass spectrometric analysis, translation reactions were conducted at 37°C for 30 min in the presence of unlabeled Asp. The reaction mixture was then diluted with an equal volume of 2 × TBS buffer [100 mM Tris-HCl (pH 7.6), 300 mM NaCl], mixed with anti-FLAG M2 affinity gel (Sigma-Aldrich), and incubated for 15 min at room temperature. The beads were washed twice with 1 × TBS buffer [50 mM Tris-HCl (pH 7.6), 150 mM NaCl] and eluted with 0.2% trifluoroacetic acid. The eluate was applied to an SPE C-tip (Nikkyo Technos), washed with 4% acetonitrile/0.5% acetic acid, and eluted with 80% acetonitrile/0.5% acetic acid containing 50%-saturated (*R*)-cyano-4-hydroxycinnamic acid. The resulting eluate was crystallized and analyzed using an UltrafleXtreme mass spectrometer (Bruker Daltonics) in reflector/positive mode, with Peptide Calibration Standard II (Bruker Daltonics) employed for external calibration.

For quantification of translated peptides by autoradiography, [^14^C]-Asp was used in place of unlabeled Asp. All model peptides contained a FLAG tag (Asp-Tyr-Lys-Asp-Asp-Asp-Asp-Lys), in which the Asp residues were radiolabeled with [^14^C]-Asp. Translation was performed at 37°C, then stopped by adding an equal volume of stop solution [0.9 M Tris-HCl (pH 8.45), 8% SDS, 30% glycerol, 0.001% xylene cyanol] and incubating at 95°C for 2 min. The samples were analyzed by 15% tricine SDS–PAGE followed by autoradiography using a Typhoon FLA 7000 imaging system (Cytiva). Absolute translation levels were determined by the radioisotope intensity of [^14^C]-Asp-labeled peptides relative to the total [^14^C]-Asp signal in the reaction mixture.

## Results

### Ribosomal incorporation of consecutive BAAs into model peptides in the presence of *E. coli* YebC or YeeN

**Figure 1.**
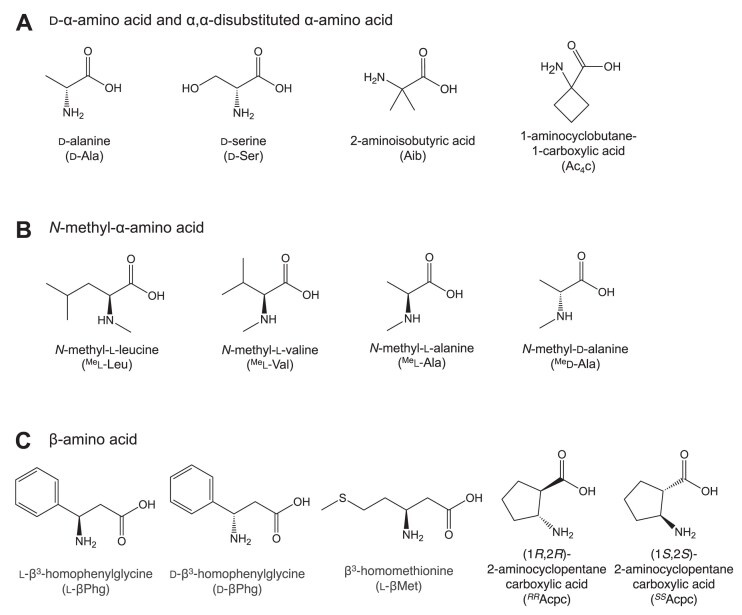
Chemical structures of backbone-altering nonproteinogenic amino acids (BAAs) tested in this study. (**A**) d-α-amino acid and α,α-disubstituted α-amino acid; (**B**) *N*-methyl-α-amino acid; and (**C**) β-amino acid.

**Figure 2.**
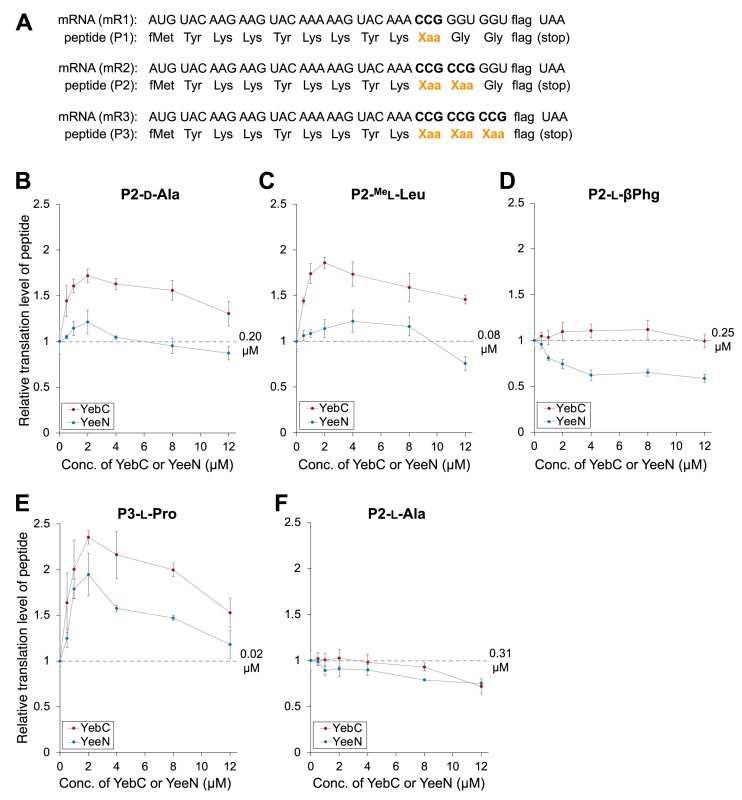
Titration of YebC and YeeN concentrations during translation of model peptides containing consecutive BAAs. (**A**) Sequences of mRNAs (mR1–3) and corresponding peptides (P1–3). “Xaa” denotes amino acids introduced at CCG codons via precharged aminoacyl-tRNAs. The amino-acid sequence of the “flag” tag is Asp–Tyr–Lys–Asp–Asp–Asp–Asp–Lys. (B–F) Quantification of relative translation levels of P2 or P3 peptides containing BAAs: P2-d-Ala (**B**), P2-^Me^l-Leu (**C**), P2-l-βPhg (**D**), P3-l-Pro (**E**), and P2-l-Ala (**F**). YebC and YeeN concentrations varied from 0 to 12 µM. Error bars represent standard deviation (SD) from three independent experiments. See also Supplementary Fig. S2A for raw Tricine-SDS-PAGE data. Absolute translation levels in the absence of YebC/YeeN are indicated to the right of the dotted lines.

To test the hypothesis that *E. coli* YebC and YeeN promote ribosomal incorporation of various BAAs in addition to l-Pro, we examined the consecutive incorporation of d-α-amino acids, *N*-methyl-l-α-amino acids, and β-amino acids into a model peptide, P2 (Figs 1, 2A). As representatives of these BAA classes, d-Ala, ^Me^l-Leu, and l-βPhg were selected. The incorporation of l-Pro (into P3) and l-Ala (into P2) served as positive and negative controls, respectively. Each amino acid was precharged onto tRNA^Pro1E2^_CGG_—a tRNA^Pro1E2^ variant bearing the CGG anticodon (Supplementary Fig. S1A)—using the flexizyme variant dFx [27, 57], and incorporated at CCG codons of the template mRNAs.

For peptide quantification, translation was performed in the presence of [^14^C]-Asp using a customized FIT system (see Supplementary Table S3 for composition). YebC and YeeN concentrations varied from 0 to 12 µM. EF-P and ABC-F were omitted from the translation system in these assays. The resulting peptides were separated by Tricine SDS–PAGE and quantified via autoradiography (Fig. 2B–F, Supplementary Fig. S2A). The absolute translation levels of P2-d-Ala, P2-^Me^l-Leu, P2-l-βPhg, P3-l-Pro, and P2-l-Ala in the absence of YebC and YeeN were 0.20 µM, 0.08 µM, 0.25 µM, 0.02 µM, and 0.31 µM, respectively. Relative translation levels in the presence of YebC and YeeN, compared to their absence, were also determined and are summarized in Fig. 2B–F.

In the translation of P2-d-Ala and P2-^Me^l-Leu, the addition of YebC significantly enhanced their translation levels within the tested YebC concentration range (0.5–12 µM) (Fig. 2B,C). The enhancement peaked at 2 µM YebC, where the relative P2-d-Ala and P2-^Me^l-Leu levels reached 1.7 and 1.9, respectively, and gradually declined at higher YebC concentrations. In contrast, YeeN was less effective, resulting in at most a 1.2-fold increase for both P2-d-Ala and P2-^Me^l-Leu translation. Moreover, adding a high concentration of YeeN (12 µM) caused a marked decrease in their relative translation levels below 1. For P2-l-βPhg translation, its relative level peaked at 1.1 with 2–8 µM YebC, whereas YeeN addition inhibited its translation (Fig. 2D). The relative P3-l-Pro level was enhanced 2.4-fold and 1.9-fold by adding 2 µM YebC or 2 µM YeeN, respectively, whereas P2-l-Ala translation was not significantly affected by either factor (Fig. 2E, F). These results indicate that YebC and YeeN can promote incorporation of these BAAs as well as l-Pro, but not l-Ala; the extent of promotion depends on the BAA type, as exemplified by the inhibition of P2-l-βPhg translation by YeeN. Given that excessively high concentrations of YebC and YeeN proved detrimental, 2 µM YebC or YeeN was used for subsequent experiments. To verify translation accuracy, the identities of the translated peptides were confirmed by MALDI-TOF MS (Supplementary Fig. S3A; P2-d-Ala, P2-^Me^l-Leu, P2-l-βPhg, P3-l-Pro, and P2-l-Ala).

**Figure 3.**
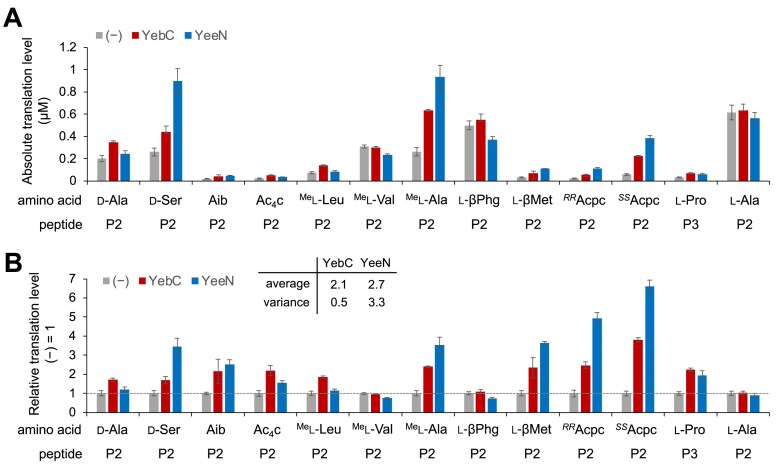
T ranslation of model peptides incorporating 13 different amino acids in the presence of YebC or YeeN. (**A, B**) Quantification of absolute (**A**) and relative (**B**) translation levels of P2 or P3 peptides. Red and blue bars indicate the addition of 2 µM YebC and 2 µM YeeN, respectively; grey bars indicate reactions performed without YebC/YeeN. Error bars represent SD from three independent experiments. See also Supplementary Fig. S2A, B for raw Tricine-SDS-PAGE data. The inset shows the average and variance of fold-enhancement across the 11 BAAs upon YebC or YeeN addition. Results for P2-d-Ala, P2-^Me^l-Leu, P2-l-βPhg, P3-l-Pro, and P2-l-Ala correspond to those in Fig. 2B–F.

To further evaluate the applicability of YebC and YeeN to diverse BAAs, 11 BAAs were tested for consecutive incorporation into P2 in the presence of 2 µM YebC or YeeN: d-Ala, d-Ser, Aib, and Ac_4_c as representatives of d-amino and α,α-disubstituted α-amino acids; ^Me^l-Leu, ^Me^l-Val, and ^Me^l-Ala as representatives of *N*-methyl-l-α-amino acids; and l-βPhg, l-βMet, *^RR^*Acpc, and *^SS^*Acpc as representatives of β-amino acids (Fig. 1). Their absolute and relative translation levels were quantified as described above (Fig. 3, Supplementary Fig. S2B). Consequently, in the incorporation of d-Ala, Ac_4_c, ^Me^l-Leu, ^Me^l-Val, and l-βPhg, the addition of YebC produced higher relative translation levels than YeeN (Fig. 3B; 1.7, 2.2, 1.9, 1.0, and 1.1, respectively), whereas the incorporation of d-Ser, Aib, ^Me^l-Ala, l-βMet, *^RR^*Acpc, and *^SS^*Acpc was more strongly promoted by YeeN than by YebC (Fig. 3B; 3.4, 2.5, 3.5, 3.7, 4.9, and 6.6, respectively). It should be noted that the relatively high translation levels of some BAAs compared to l-Ala are due to the use of tRNA^Pro1E2^, which is optimized for BAAs but can be suboptimal for canonical l-amino acids. For YebC, no significant inhibitory effect was observed for any of the 11 BAAs, whereas YeeN inhibited ^Me^l-Val and l-βPhg incorporation (Fig. 3B; 0.8 and 0.7). The average enhancement effects of YebC and YeeN for these 11 BAAs were 2.1-fold and 2.7-fold, respectively, with variances of 0.5 and 3.3 (Fig. 3B, inset). These values indicate that, on average, YeeN exhibited greater enhancement effects than YebC, with a broader range of variation (0.7−6.6). In contrast, YebC showed a more modest and stable effect, ranging from 1.0 to 3.8. These findings suggest that the choice between YebC and YeeN should be tailored to the specific type of BAA being incorporated.

### Competition between YebC and YeeN and their synergy with EF-P and Uup

**Figure 4.**
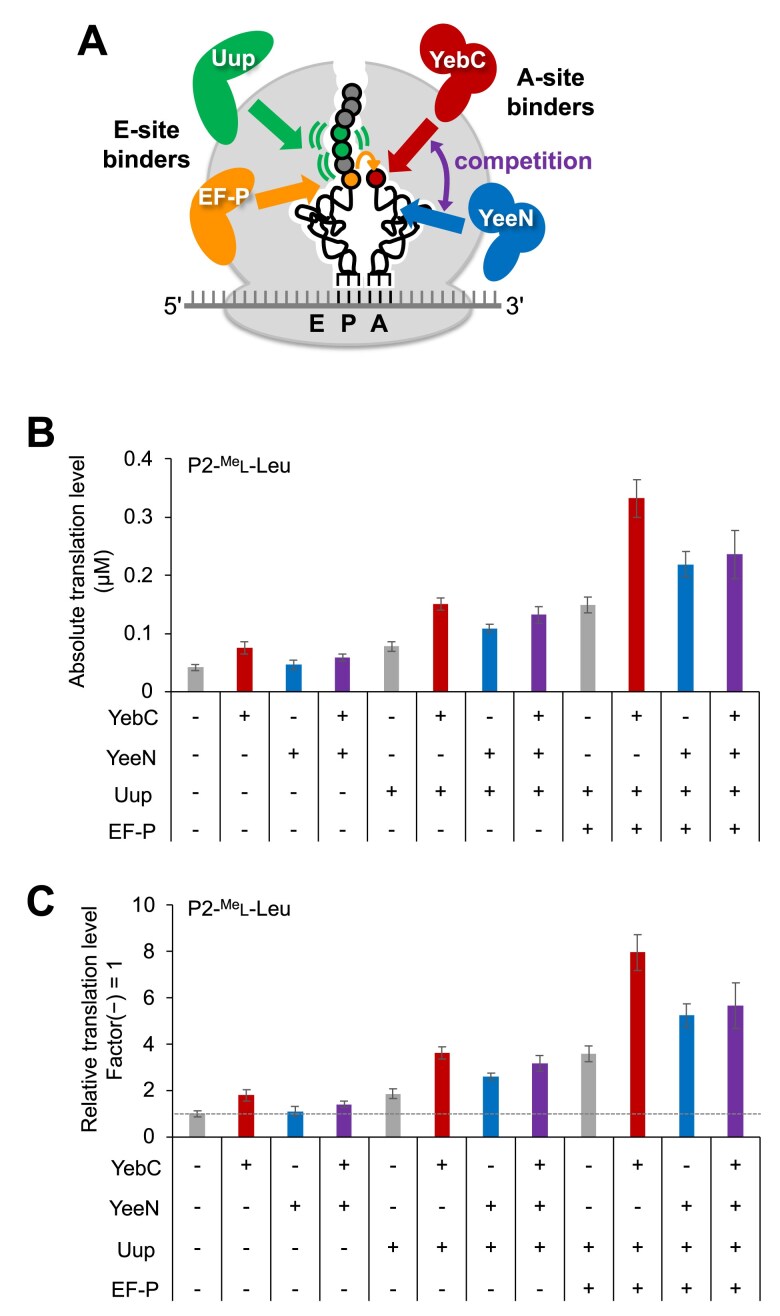
Combined effects of YebC, YeeN, Uup, and EF-P on translation of P2-^Me^l-Leu. (**A**) Binding sites of the four protein factors. E-site binders Uup and EF-P act at different stages and therefore do not compete, whereas A-site binders YebC and YeeN compete for the same binding site. (**B, C**) Quantification of absolute (**B**) and relative (**C**) translation levels of P2-^Me^l-Leu. Final concentrations of YebC, YeeN, Uup, and EF-P were 2, 2, 1, and 5 µM, respectively. Error bars represent SD from three independent experiments. See also Supplementary Fig. S2C for raw Tricine-SDS-PAGE data.

To further enhance the consecutive BAA incorporation, we next examined the effects of introducing EF-P and ABC-F factors into the translation system in addition to YebC and YeeN. In this study, Uup was selected as a representative ABC-F protein. Given that YebC and YeeN are both putative A-site binders, we hypothesized that they might compete for the same binding site when introduced simultaneously, thereby affecting BAA incorporation. In contrast, since EF-P and Uup are E-site binders, they are unlikely to compete with A-site-binding YebC or YeeN. We recently reported that EF-P and Uup do not compete with each other for E-site binding [41]. This difference likely reflects their distinct binding contexts: EF-P binds to the E site when peptidyl-BAA-tRNA^Pro1E2^ occupies the P site to promote peptidyl transfer, whereas Uup associates with the E site to alleviate translation arrest caused by nascent peptides containing multiple BAAs. Under the latter condition, the BAAs have already been incorporated, and the tRNA^Pro1E2^ has exited, meaning EF-P no longer occupies the E site and therefore does not compete with Uup. Thus, potential competition among these four factors is expected only between YebC and YeeN (Fig. 4A, indicated by the purple double-headed arrow).

To verify this hypothesis, we quantified the translation levels of P2-^Me^l-Leu using various combinations of YebC, YeeN, EF-P, and Uup (Fig. 4B,C, Supplementary Fig. S2C). The concentrations of these proteins were 2 µM, 2 µM, 5 µM, and 1 µM, respectively. The absolute translation level of P2-^Me^l-Leu in the absence of these factors was estimated to be 0.04 µM, which was set as 1 for calculating relative translation levels in the presence of the factors. The addition of YebC or YeeN enhanced the relative P2-^Me^l-Leu level to 1.8 and 1.1, respectively. However, when both YebC and YeeN were added together, the relative level decreased to 1.4—lower than that achieved by YebC alone—indicating that YeeN competitively inhibits YebC activity. In contrast, Uup alone increased the relative level to 1.9. Its co-introduction with YebC further elevated the yield to 3.6, demonstrating that these two factors function non-competitively. Similarly, Uup and YeeN showed no competition, with their combination yielding a level of 2.6. However, when both YebC and YeeN were added together with Uup, the relative level decreased to 3.2, again supporting competition between YebC and YeeN. When EF-P was introduced with Uup, the relative P2-^Me^l-Leu level rose to 3.6, notably higher than Uup alone (1.9), indicating no competition between EF-P and Uup. Furthermore, the addition of YebC, along with EF-P and Uup, further enhanced the relative level to 8.0. Likewise, introducing YeeN together with EF-P and Uup produced a higher enhancement (5.2). In contrast, when all four factors—YebC, YeeN, EF-P, and Uup—were added, the relative P2-^Me^l-Leu level decreased to 5.7, lower than that observed with YebC, EF-P, and Uup, again indicating competition between YebC and YeeN. These results support the hypothesis that EF-P, Uup, and YebC act independently on ribosome binding, whereas YebC and YeeN compete. Because EF-P, Uup, and YebC each enhance P2-^Me^l-Leu translation by approximately twofold and act synergistically, the combined addition of all three resulted in a substantial eightfold overall enhancement (2 × 2 × 2).

### Sequence dependence of YeeN-mediated enhancement of ^Me^l-Ala incorporation

**Figure 5.**
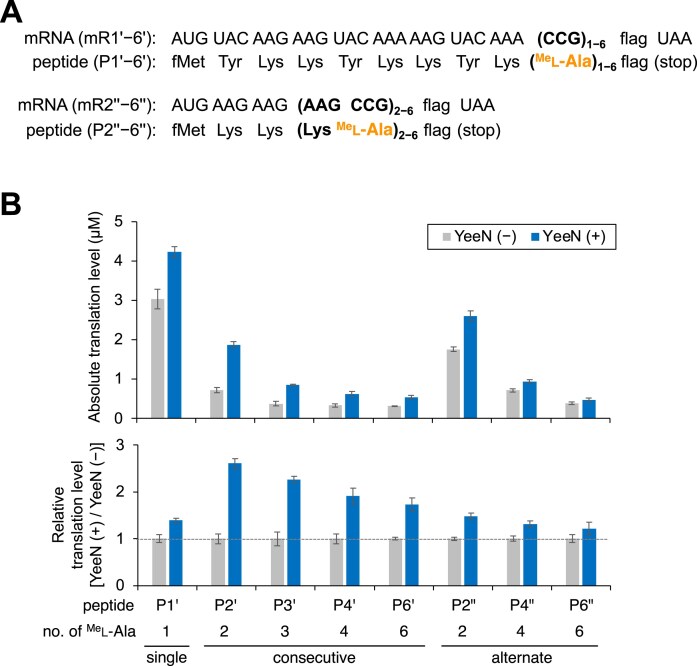
Sequence dependence of YeeN-mediated translation enhancement during ^Me^l-Ala incorporation. (**A**) Sequences of mRNAs (mR1′–6′ and mR2″–6″) and corresponding peptides (P1′–6′ and P2″–6″) used in this experiment. The amino acid sequence of the “flag” tag is Asp–Tyr–Lys–Asp–Asp–Asp–Asp–Lys. (**B**) Quantification of absolute and relative translation levels of model peptides containing ^Me^l-Ala. Error bars represent SD from three independent experiments. See also Supplementary Fig. S2D for raw Tricine-SDS-PAGE data.

Next, we examined how the peptide sequence influences the YeeN effect, using ^Me^l-Ala as a model BAA. We designed template mRNAs—mR1′, mR2′−6′, and mR2″−6″—for single, consecutive, and alternate incorporations of ^Me^l-Ala, respectively (Fig. 5A). ^Me^l-Ala was precharged onto tRNA^Pro1E2^_CGG_ and introduced at CCG codons of these mRNAs to synthesize P1′, P2′−6′, and P2″−6″. Absolute and relative translation levels were quantified in the presence or absence of 2 µM YeeN (Fig. 5B, Supplementary Fig. S2D), where the peptide level without YeeN was set as 1 for relative comparison. Compared with the single incorporation of ^Me^l-Ala into P1′, consecutive incorporation into P2′−6′ proved more challenging. Accordingly, absolute translation levels of P1′−6′ decreased as the number of ^Me^l-Ala residues increased [from 3.0 µM to 0.3 µM in YeeN (−) and from 4.2 µM to 0.5 µM in YeeN (+)]. Upon addition of YeeN, the P1′-^Me^l-Ala level increased 1.3-fold, while the P2′-^Me^l-Ala level rose 2.6-fold, indicating that peptide bond formation between consecutive ^Me^l-Ala residues in P2″ is inefficient but strongly promoted by YeeN. However, for longer consecutive incorporations (P3′−6′), the enhancement gradually declined from 2.3- to 1.7-fold.

These observations suggest that bond formation between the first and second ^Me^l-Ala is the most difficult step, requiring YeeN for efficient incorporation, whereas subsequent additions proceed more readily, diminishing YeeN’s influence. In alternate incorporations (P2″, P4″, and P6″), relative translation levels with YeeN were 1.5, 1.3, and 1.2, respectively—significantly lower than those of P2′, P4′, and P6′ (2.6, 1.9, and 1.7). Thus, alternate ^Me^l-Ala incorporation is less hindered by the ribosomal machinery than consecutive incorporation, and YeeN’s stimulatory effect is correspondingly reduced. To confirm the fidelity of translation, we performed MALDI-TOF MS analysis on the most challenging case—the six-residue consecutive incorporation (P6′-^Me^l-Ala). The target peptide was correctly detected in both the presence and absence of 2 µM YeeN, without any detectable misincorporation (Supplementary Fig. S3B).

### Effects of YebC and YeeN in translation initiation and early elongation

**Figure 6.**
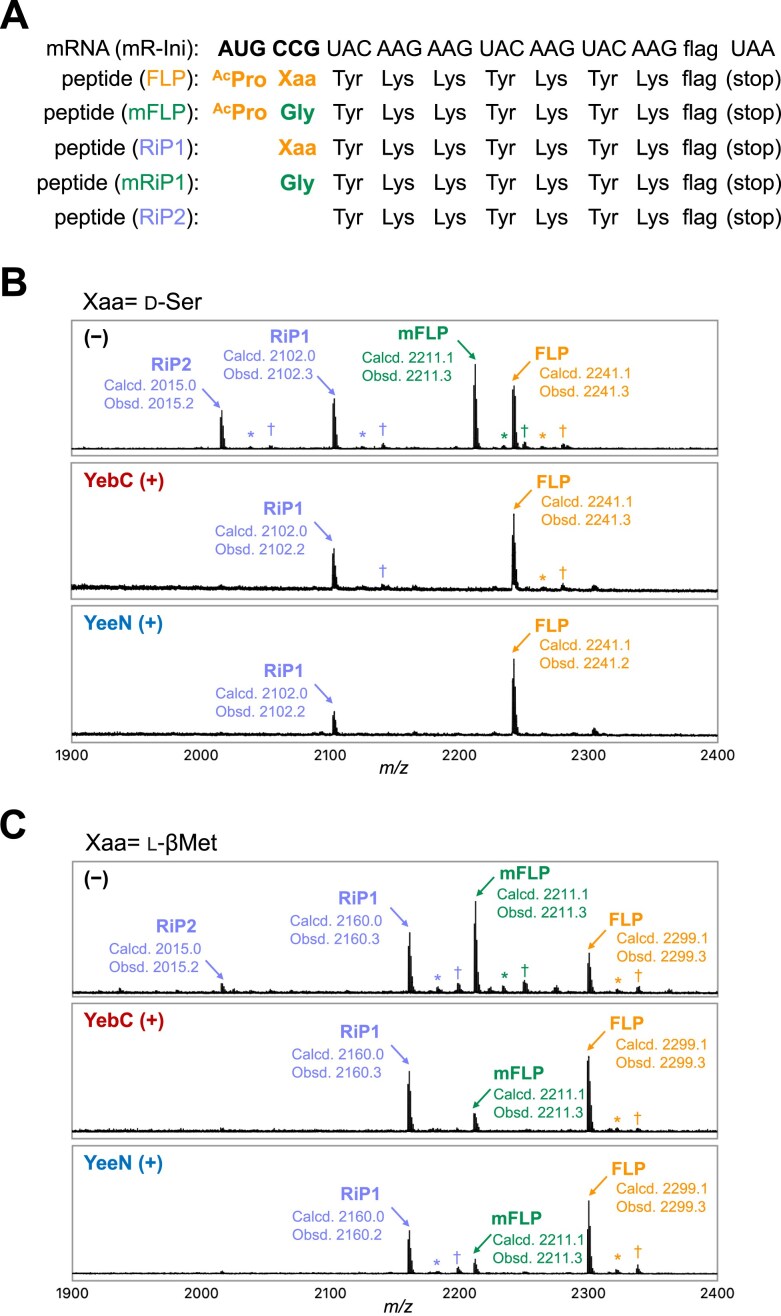
Effects of YebC and YeeN on translation of model peptides bearing consecutive BAAs at the *N*-terminus. (**A**) Sequence of mRNA (mR-Ini) and corresponding peptides used in this experiment. The amino acid sequence of the “flag” tag is Asp–Tyr–Lys–Asp–Asp–Asp–Asp–Lys. FLP denotes the desired full-length peptide; other bands correspond to drop-off reinitiation and/or misincorporation products. mFLP and mRiP1 contain Gly misincorporation at the CCG codon. (**B, C**) MALDI-TOF MS analysis of translation products incorporating d-Ser (**B**) or l-βMet (**C**) at the CCG codon. “Calcd.” and “Obsd.” denote calculated and observed *m/z* values of the desired peptides, respectively. * and † indicate Na^+^ and K^+^ adducts.

We next examined the effects of YebC and YeeN on translation initiation and the subsequent incorporation of the first elongator amino acid. A model mRNA, mR-Ini, was designed such that the initiator AUG codon encodes *N*-acetyl-l-proline (^Ac^Pro) and the second CCG codon encodes either d-Ser or l-βMet to synthesize a full-length peptide, FLP (Fig. 6A). The initiator ^Ac^Pro was precharged onto tRNA^fMet^_CAU_, while the elongators d-Ser and l-βMet were precharged onto tRNA^Pro1E2^_CGG_.

We previously reported that ^Ac^Pro is a poor initiator substrate that frequently triggers drop-off reinitiation events [58, 59]. Consequently, a truncated peptide, RiP1, lacking the N-terminal ^Ac^Pro, is expected as a byproduct. When d-Ser was incorporated at the second position, both FLP and RiP1 were observed as expected (Fig. 6B). Misincorporation of Gly in place of d-Ser (yielding mFLP) and drop-off reinitiation at d-Ser (yielding RiP2) also occurred in the absence of YebC and YeeN, likely reflecting the inherent difficulty of d-Ser incorporation. However, the addition of 2 µM YebC or YeeN promoted efficient d-Ser incorporation, eliminating mFLP and RiP2 [Fig. 6B, YebC (+) and YeeN (+)]. The relative RiP1 level was likewise reduced. Similarly, when l-βMet was incorporated at the second position, YebC or YeeN markedly decreased mFLP and RiP2 levels by enhancing l-βMet incorporation (Fig. 6C), thereby increasing the relative FLP yield. These findings demonstrate that YebC and YeeN effectively facilitate the incorporation of inefficient substrates at the peptide N-terminus, suppressing undesired bypass events during the early stages of translation.

### Ribosomal synthesis of model macrocyclic peptides bearing multiple BAAs in the presence of YebC or YeeN

**Figure 7.**
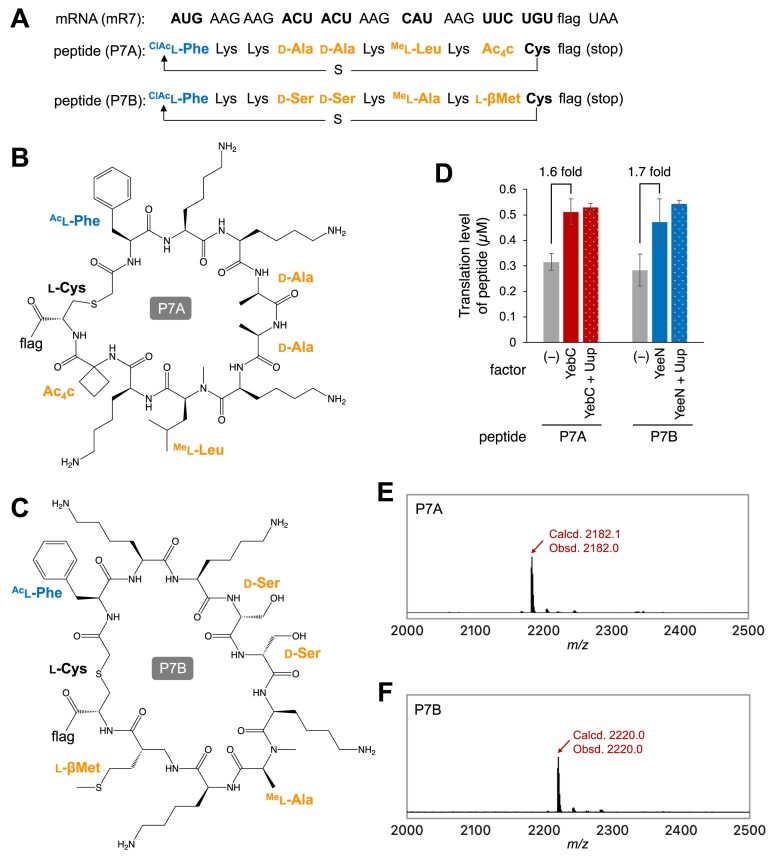
R ibosomal synthesis of model macrocyclic peptides bearing multiple BAAs in the presence of YebC or YeeN. (**A**) Sequences of mRNA (mR7) and corresponding peptides (P7A and P7B). (**B, C**) Chemical structures of the model macrocyclic peptides P7A and P7B. The sulfhydryl group of Cys spontaneously reacts with the N-terminal ClAc group to form a thioether bond, generating a macrocyclic scaffold. The amino acid sequence of the “flag” tag is Asp–Tyr–Lys–Asp–Asp–Asp–Asp–Lys. (**D**) Quantification of translated peptides P7A and P7B. Error bars represent SD from three independent experiments. See also Supplementary Fig. S2E for raw Tricine-SDS-PAGE data. (**E, F**) MALDI-TOF MS identification of translated peptides P7A (**E**) and P7B (**F**). Translation was performed in the presence of 2 µM YebC (**E**) or 2 µM YeeN (**F**). Red arrows denote monovalent ions ([M + H]^+^) of the desired products. “Calcd.” and “Obsd.” indicate calculated and observed *m/z* values, respectively.

One aim of this study was to achieve the efficient translation of macrocyclic peptides containing multiple BAAs, such as d-α-, *N*-methyl-l-α-, and β-amino acids, by leveraging YebC and YeeN. Here, we demonstrate the ribosomal synthesis of two model macrocyclic peptides, P7A and P7B, each containing four BAAs and synthesized from the same mRNA, mR7 (Fig. 7A–C). For macrocyclization, ^ClAc^l-Phe was precharged onto tRNA^iniP^_CAU_ and introduced at the initiator AUG codon. This engineered initiator tRNA features a specific D-arm motif that recruits EF-P to promote peptide bond formation [60]. The chloroacetyl group of ^ClAc^l-Phe spontaneously reacts with the thiol group of a downstream Cys residue to form a thioether linkage, completing macrocyclization [61]. During the translation of P7A, d-Ala, ^Me^l-Leu, and Ac_4_c were precharged onto tRNA^Pro1E2^_GGU_, tRNA^Pro1E2^_GUG_, and tRNA^Pro1E2^_GAA_ and incorporated at the ACU, CAU, and UUC codons of mR7, respectively. For P7B, d-Ser, ^Me^l-Ala, and l-βMet were precharged on the same tRNAs and introduced at the corresponding codons.

Based on our earlier findings that YebC preferentially supports the substrates used in P7A (d-Ala, ^Me^l-Leu, and Ac_4_c), whereas YeeN is more effective for those in P7B (d-Ser, ^Me^l-Ala, and l-βMet), we utilized YebC for P7A translation and YeeN for P7B (Fig. 7D–F; Supplementary Fig. S2E). As a result, YebC and YeeN increased the yields of P7A and P7B by 1.6- and 1.7-fold, respectively, demonstrating their utility in the ribosomal synthesis of macrocyclic peptides enriched with BAAs (Fig. 7D). We also examined the effects of adding Uup in combination with YebC (for P7A) or YeeN (for P7B); however, no significant further increase in yield was observed. This lack of additional enhancement likely suggests that these specific sequences do not induce substantial ribosomal stalling via interactions between the nascent peptide and the exit tunnel, thereby limiting the potential contribution of Uup to translation efficiency; consequently, YebC or YeeN alone was sufficient to facilitate their translation effectively. Finally, the translation fidelity of P7A and P7B in the presence of YebC or YeeN was verified by MALDI-TOF MS analysis, which showed the expected products as clean, dominant peaks (Fig. 7E, F).

### Evaluation of *A. macleodii* YebC and YeeN in combination with the *A. macleodii* ribosome

We recently reported that the *A. macleodii* ribosome incorporates d-α-amino acids, d-β-amino acids, α,α-disubstituted α-amino acids, and *N*-methyl-d-α-amino acids more efficiently than the *E. coli* ribosome [54]. For example, the incorporation of six consecutive d-Ser residues and two consecutive Ac_4_c residues was 6.1-fold and 7.3-fold more efficient, respectively, with the *A. macleodii* ribosome. These experiments were conducted using a heterologous translation system composed of the *A. macleodii* ribosome and *E. coli* translation components, including initiation, elongation, and release factors, aminoacyl-tRNA synthetases, and tRNAs. We also compared the activities of *A. macleodii* EF-P and Uup with those of *E. coli*, finding that the *A. macleodii* factors exhibited significantly higher activities in this system. These results motivated us to evaluate *A. macleodii* YebC and YeeN in combination with the *A. macleodii* ribosome.

**Figure 8.**
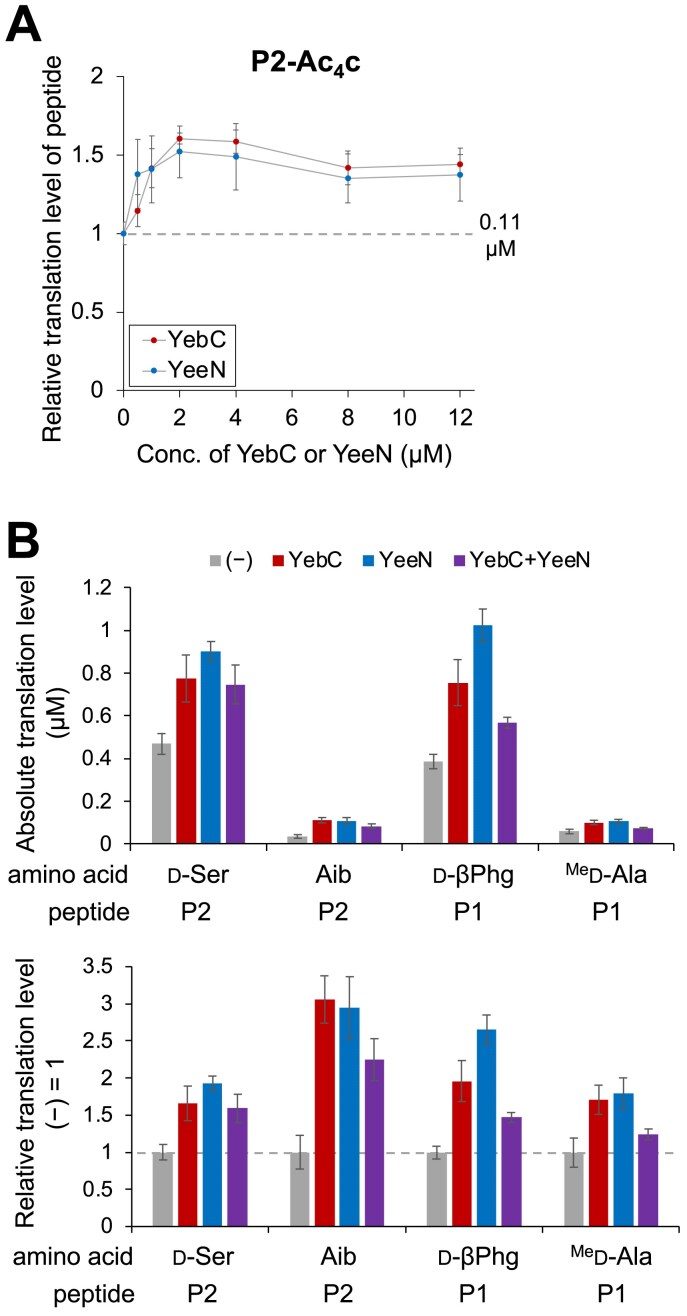
E valuation of *Alteromonas macleodii* YebC and YeeN activities in BAA incorporation. (**A**) Titration of *A. macleodii* YebC and YeeN concentrations in translation of P2-Ac_4_c. Translation was performed using the *A. macleodii* ribosome in place of the *E. coli* ribosome, together with *E. coli* translation factors except for *A. macleodii* YebC and YeeN. (**B**) Quantification of absolute and relative translation levels of P1 and P2 peptides. Red and blue bars indicate the addition of 2 µM YebC and 2 µM YeeN, respectively; purple bars indicate the addition of both; grey bars represent reactions without YebC/YeeN. Error bars represent SD from three independent experiments. See also Supplementary Fig. S2F, G for raw Tricine-SDS-PAGE data.

We first evaluated the translation of P2-Ac_4_c in the presence of 0–12 µM *A. macleodii* YebC or YeeN (Fig. 8A; Supplementary Fig. S2F). The enhancement effect peaked at 2 µM for both factors, where the relative P2-Ac_4_c levels reached 1.6 and 1.5, respectively, and gradually declined at higher concentrations. Thus, 2 µM *A. macleodii* YebC or YeeN was used in subsequent experiments.

The absolute P2-Ac_4_c levels achieved with *A. macleodii* YebC or YeeN in combination with the *A. macleodii* ribosome were 0.18 and 0.17 µM, respectively—substantially higher than those obtained using *E. coli* YebC or YeeN with the *E. coli* ribosome (Fig. 3A, 0.05 µM and 0.04 µM, respectively). These results highlight the advantage of *A. macleodii* YebC, YeeN, and ribosomes in Ac_4_c incorporation.

To further assess the enhancement effects of *A. macleodii* YebC and YeeN, we next evaluated translation of P2-d-Ser, P2-Aib, P1-d-βPhg, and P1-^Me^d-Ala (Fig. 8B; Supplementary Fig. S2G). Incorporation of d-βPhg and ^Me^d-Ala is too inefficient for consecutive introduction into P2, so their single incorporation into P1 was examined instead. The addition of 2 µM YebC increased translation levels of these four peptides by 1.7-, 3.1-, 2.0-, and 1.7-fold, respectively, while 2 µM YeeN enhanced them by 1.9-, 2.9-, 2.6-, and 1.8-fold. However, when both YebC and YeeN were added simultaneously, the enhancement effects decreased to 1.6-, 2.2-, 1.5-, and 1.2-fold, respectively, suggesting that *A. macleodii* YebC and YeeN compete for A-site binding. Finally, translation accuracy for P2-Ac_4_c, P2-d-Ser, P2-Aib, P1-d-βPhg, and P1-^Me^d-Ala was confirmed by MALDI-TOF MS analysis, where the expected products were clearly observed as dominant peaks (Supplementary Fig. S3C).

## Discussion

It has been recently proposed that YebC and YeeN are putative A-site binding factors involved in promoting consecutive Pro incorporations in nature [51]. Although their exact mechanism remains elusive, our results suggest that their mode of facilitating BAA incorporation parallels their role in Pro translation. One plausible scenario is that YebC/YeeN transiently sample an A-site–proximal position during the elongation cycle, either rearranging the 23S rRNA surrounding the A site or directly engaging the A-site aminoacyl–tRNA to induce a more reactive geometry. This model is supported by a recent structural study of the human mitochondrial homolog TACO1 bound to the mitochondrial ribosome [62]. The structure reveals that TACO1 interacts not only with the rRNA but also directly stabilizes the A-site tRNA by engaging its acceptor stem, which likely induces a more reactive geometry for peptide bond formation within the PTase center.　

Such an A-site-focused mechanism is fundamentally distinct from those of EF-P and ABC-F proteins, which bind to the E-site. EF-P stabilizes the P-site peptidyl-Pro-tRNA near the PTase center, thereby preventing ribosome stalling and accelerating the otherwise slow peptide bond formation at polyproline motifs [43, 44, 63]. ABC-F proteins remodel the P-site and PTase center to rescue stalled ribosomes [49, 64, 65]. Consistent with the structural finding that TACO1 functions at the A-site, our biochemical data show that YebC or YeeN can be used synergistically with EF-P and Uup. For instance, the simultaneous addition of YebC, EF-P, and Uup enhanced ^Me^l-Leu incorporation approximately eightfold (Fig. 4C). Integrating these multiple factors and species-optimized ribosomes, such as the *A. macleodii* system, provides a superior platform for synthesizing BAA-rich peptides.

However, the efficacy of these factors is highly concentration-dependent. We observed that excessive concentrations of YebC or YeeN (> 2 µM) diminished their enhancing effects and, in some cases, inhibited translation. This observation finds a structural rationale in the TACO1–ribosome complex, where TACO1 was shown to compete with EF-Tu for A-site occupancy. While TACO1 may promote EF-Tu turnover by facilitating its dissociation from the A site, an excess of the factor could conversely interfere with the fundamental function of EF-Tu. Thus, YebC and YeeN must be maintained at optimal concentrations to avoid interfering with essential A-site-binding factors such as EF-Tu and EF-G. Furthermore, our results indicate that YebC and YeeN compete with each other, highlighting the importance of selecting the single optimal factor for a specific BAA substrate.

The substrate-dependent preferences of YebC and YeeN likely reflect their ability to optimize the orientation of the A-site aminoacyl-tRNA. We hypothesize that these factors facilitate peptide bond formation by repositioning the reactive amino group of the A-site tRNA within the PTase center. Given that YebC promotes consecutive Pro incorporation more effectively than YeeN (Fig. 2E), YebC may possess a superior ability to realign the Pro-tRNA into an ideal reactive geometry. In contrast, for various BAAs, differences in backbone structures and side-chains likely lead to distinct initial positions of the amino group. Furthermore, subtle structural differences between YebC and YeeN may influence how each factor interacts with the A-site tRNA or the surrounding rRNA, thereby altering the specific direction and magnitude of tRNA repositioning. Recent studies on diverse structural classes of BAAs, including β-amino and β-hydroxy acids, have shown that reactive substrates favor a conformational space where the aminoacyl-tRNA nucleophile is located within 4 Å of the peptidyl-tRNA carbonyl electrophile with a Bürgi–Dunitz angle of 76–115° [29, 66]. We therefore speculate that YebC and YeeN promote peptide bond formation not merely by stabilizing A-site BAA-tRNA, but by facilitating its transition from an accommodated yet suboptimal state to a catalytically competent state. In this process, these factors may guide the reactive amino group of diverse BAAs into such an optimal arrangement. Consequently, the observed variation in effectiveness suggests that either YebC or YeeN may be better suited to optimizing the orientation of a specific BAA substrate.

Finally, we achieved the efficient ribosomal synthesis of macrocyclic peptides containing multiple d-α-, α,α-disubstituted-α-, *N*-methyl-l-α-, and β-amino acids in the presence of YebC or YeeN (Fig. 7). These BAAs induce unique and stable peptide conformations, such as turns and helices [1, 3–5, 7, 13, 14], while macrocyclization further rigidifies the peptide scaffold. Such rigidified macrocyclic peptides bearing BAAs often exhibit superior target-binding affinity, inhibitory potency, proteolytic stability, and membrane permeability [9, 12–14, 21, 67], positioning them as highly attractive candidates for therapeutic development. Ribosomal synthesis offers a distinct advantage over chemical synthesis by enabling the rapid generation of diverse random peptide libraries through mRNA template diversification, which are compatible with high-throughput screening platforms like the RaPID platform [22]. Integrating YebC-family factors and species-optimized ribosomes into these systems will facilitate the construction of libraries with higher densities and broader combinations of BAAs. This expansion of accessible conformational and functional space will ultimately accelerate the discovery of potent bioactive peptides against a wide range of disease-related targets.

## Supplementary Material

gkag617_Supplemental_Files

## Data Availability

The data underlying this research are available in the article and in its online supplementary material.

## References

[1] Karle IL, Awasthi SK, Balaram P. A designed β-hairpin peptide in crystals. Proc Natl Acad Sci USA. 1996;93:8189–93. 10.1073/pnas.93.16.8189. 8710845 PMC38644

[2] Kaul R, Balaram P. Stereochemical control of peptide folding. Bioorg Med Chem. 1999;7:105–17. 10.1016/S0968-0896(98)00221-1. 10199661

[3] Aravinda S, Shamala N, Rajkishore R et al. A crystalline β-hairpin peptide nucleated by a type I' Aib-D-Ala β-turn: evidence for cross-strand aromatic interactions. Angew Chem Int Ed. 2002;41:3863–5. 10.1002/1521-3773(20021018)41:20<3863::AID-ANIE3863>3.0.CO;2-A. 12386872

[4] Appella DH, Christianson LA, Karle IL et al. β-peptide foldamers: robust helix formation in a new family of β-amino acid oligomers. J Am Chem Soc. 1996;118:13071–2. 10.1021/ja963290l.

[5] Schumann F, Müller A, Koksch M et al. Are β-amino acids γ-turn mimetics? Exploring a new design principle for bioactive cyclopeptides. J Am Chem Soc. 2000;122:12009–10. 10.1021/ja0016001.

[6] Guthohrlein EW, Malesevic M, Majer Z et al. Secondary structure inducing potential of β-amino acids: torsion angle clustering facilitates comparison and analysis of the conformation during MD trajectories. Biopolymers. 2007;88:829–39. 10.1002/bip.20859. 17922495

[7] Kawai M, Malla TR, Chan HTH et al. RaPID discovery of cell-permeable helical peptide inhibitors containing cyclic β-amino acids against SARS-CoV-2 main protease. RSC Chem Biol. 2025;6:1089–99. 10.1039/D5CB00021A. 40406165 PMC12093385

[8] Hintermann T, Gademann K, Jaun B et al. γ-peptides forming more stable secondary structures than α-peptides: synthesis and helical NMR-solution structure of the γ-hexapeptide analog of H-(Val-Ala-Leu)_2_-OH. Helv Chim Acta. 2005;81:983–1002. 10.1002/hlca.19980810514.

[9] Rader AFB, Reichart F, Weinmuller M et al. Improving oral bioavailability of cyclic peptides by N-methylation. Bioorg Med Chem. 2018;26:2766–73. 10.1016/j.bmc.2017.08.031. 28886995

[10] Molhoek EM, van Dijk A, Veldhuizen EJ et al. Improved proteolytic stability of chicken cathelicidin-2 derived peptides by D-amino acid substitutions and cyclization. Peptides. 2011;32:875–80. 10.1016/j.peptides.2011.02.017. 21376095

[11] Feng Z, Xu B. Inspiration from the mirror: d-amino acid containing peptides in biomedical approaches. Biomol Concepts. 2016;7:179–87. 10.1515/bmc-2015-0035. 27159920 PMC5316480

[12] Imanishi S, Katoh T, Yin Y et al. *In vitro* selection of macrocyclic D/L-hybrid peptides against human EGFR. J Am Chem Soc. 2021;143:5680–4. 10.1021/jacs.1c02593. 33822597

[13] Sigal M, Egner M, Okada C et al. *De novo* discovery of α,α-disubstituted α-amino acid-containing α-helical peptides as competitive PPARγ PPI inhibitors. J Am Chem Soc. 2025;147:42607–17. 41188059 10.1021/jacs.5c13803 PMC12637311

[14] Katoh T, Sengoku T, Hirata K et al. Ribosomal synthesis and *de novo* discovery of bioactive foldamer peptides containing cyclic β-amino acids. Nat Chem. 2020;12:1081–8. 10.1038/s41557-020-0525-1. 32839601

[15] Katoh T, Suga H. In vitro selection of foldamer-like macrocyclic peptides containing 2-aminobenzoic acid and 3-aminothiophene-2-carboxylic acid. J Am Chem Soc. 2022;144:2069–72. 10.1021/jacs.1c12133. 35099961

[16] Wakabayashi R, Kawai M, Katoh T et al. *In vitro* selection of macrocyclic α/β^3^-peptides against human EGFR. J Am Chem Soc. 2022;144:18504–10. 10.1021/jacs.2c07624. 36173923 PMC9563295

[17] Miura T, Malla TR, Owen CD et al. *In vitro* selection of macrocyclic peptide inhibitors containing cyclic γ^2,4^-amino acids targeting the SARS-CoV-2 main protease. Nat Chem. 2023;15:998–1005. 10.1038/s41557-023-01205-1. 37217786 PMC10322702

[18] Miura T, Lee KJ, Katoh T et al. *In vitro* selection of macrocyclic L-α/D-α/β/γ-hybrid peptides targeting IFN-γ/IFNGR1 protein–protein interaction. J Am Chem Soc. 2024;146:17691–9. 10.1021/jacs.4c01979. 38888290 PMC11229689

[19] Miura T, Malla TR, Brewitz L et al. Cyclic β^2,3^-amino acids improve the serum stability of macrocyclic peptide inhibitors targeting the SARS-CoV-2 main protease. Bull Chem Soc Jpn. 2024;97:uoae018. 10.1093/bulcsj/uoae018. 38828441 PMC11141402

[20] Yamagishi Y, Shoji I, Miyagawa S et al. Natural product-like macrocyclic N-methyl-peptide inhibitors against a ubiquitin ligase uncovered from a ribosome-expressed de novo library. Chem Biol. 2011;18:1562–70. 10.1016/j.chembiol.2011.09.013. 22195558

[21] van Neer RHP, Dranchak PK, Liu L et al. Serum-stable and selective backbone-N-methylated cyclic peptides that inhibit prokaryotic glycolytic mutases. ACS Chem Biol. 2022;17:2284–95. 10.1021/acschembio.2c00403. 35904259 PMC9900472

[22] Katoh T, Goto Y, Suga H. *In vitro* selection of thioether-closed macrocyclic peptide ligands by means of the RaPID system. Methods Mol Biol. 2022;2371:247–59. 34596852 10.1007/978-1-0716-1689-5_13

[23] Tan Z, Forster AC, Blacklow SC et al. Amino acid backbone specificity of the Escherichia coli translation machinery. J Am Chem Soc. 2004;126:12752–3. 10.1021/ja0472174. 15469251

[24] Subtelny AO, Hartman MC, Szostak JW. Ribosomal synthesis of N-methyl peptides. J Am Chem Soc. 2008;130:6131–6. 10.1021/ja710016v. 18402453 PMC2728122

[25] Dedkova LM, Fahmi NE, Golovine SY et al. Enhanced D-amino acid incorporation into protein by modified ribosomes. J Am Chem Soc. 2003;125:6616–7. 10.1021/ja035141q. 12769555

[26] Dedkova LM, Fahmi NE, Paul R et al. β-puromycin selection of modified ribosomes for *in vitro* incorporation of β-amino acids. Biochemistry. 2011;51:401–15. 10.1021/bi2016124. 22145951

[27] Goto Y, Katoh T, Suga H. Flexizymes for genetic code reprogramming. Nat Protoc. 2011;6:779–90. 10.1038/nprot.2011.331. 21637198

[28] Dunkelmann DL, Piedrafita C, Dickson A et al. Adding α,α-disubstituted and β-linked monomers to the genetic code of an organism. Nature. 2024;625:603–10. 10.1038/s41586-023-06897-6. 38200312 PMC10794150

[29] Hamlish NX, Abramyan AM, Shah B et al. Incorporation of multiple β^2^-hydroxy acids into a protein in vivo using an orthogonal aminoacyl-tRNA synthetase. ACS Cent Sci. 2024;10:1044–53. 10.1021/acscentsci.3c01366. 38799653 PMC11117724

[30] Pavlov MY, Watts RE, Tan Z et al. Slow peptide bond formation by proline and other N-alkylamino acids in translation. Proc Natl Acad Sci USA. 2009;106:50–4. 10.1073/pnas.0809211106. 19104062 PMC2629218

[31] Fujino T, Goto Y, Suga H et al. Reevaluation of the D-amino acid compatibility with the elongation event in translation. J Am Chem Soc. 2013;135:1830–7. 10.1021/ja309570x. 23301668

[32] Melnikov SV, Khabibullina NF, Mairhofer E et al. Mechanistic insights into the slow peptide bond formation with D-amino acids in the ribosomal active site. Nucleic Acids Res. 2019;47:2089–100. 10.1093/nar/gky1211. 30520988 PMC6393236

[33] Fujino T, Goto Y, Suga H et al. Ribosomal synthesis of peptides with multiple β-amino acids. J Am Chem Soc. 2016;138:1962–9. 10.1021/jacs.5b12482. 26807980

[34] Katoh T, Tajima K, Suga H. Consecutive elongation of D-amino acids in translation. Cell Chem Biol. 2017;24:46–54. 10.1016/j.chembiol.2016.11.012. 28042044

[35] Liljeruhm J, Wang J, Kwiatkowski M et al. Kinetics of D-amino acid incorporation in translation. ACS Chem Biol. 2019;14:204–13. 10.1021/acschembio.8b00952. 30648860

[36] Iwane Y, Kimura H, Katoh T et al. Uniform affinity-tuning of N-methyl-aminoacyl-tRNAs to EF-Tu enhances their multiple incorporation. Nucleic Acids Res. 2021;49:10807–17. 10.1093/nar/gkab288. 33997906 PMC8565323

[37] Katoh T, Suga H. Ribosomal incorporation of negatively charged D-α- and N-methyl-L-α-amino acids enhanced by EF-Sep. Phil Trans R Soc B Biol Sci. 2023;378:20220038. 10.1098/rstb.2022.0038. PMC9835608 36633283

[38] Cruz-Navarrete FA, Griffin WC, Chan YC et al. β-amino acids reduce ternary complex stability and alter the translation elongation mechanism. ACS Cent Sci. 2024;10:1262–75. 10.1021/acscentsci.4c00314. 38947208 PMC11212133

[39] Katoh T, Iwane Y, Suga H. Logical engineering of D-arm and T-stem of tRNA that enhances D-amino acid incorporation. Nucleic Acids Res. 2017;45:12601–10. 10.1093/nar/gkx1129. 29155943 PMC5728406

[40] Englander MT, Avins JL, Fleisher RC et al. The ribosome can discriminate the chirality of amino acids within its peptidyl-transferase center. Proc Natl Acad Sci USA. 2015;112:6038–43. 10.1073/pnas.1424712112. 25918365 PMC4434717

[41] Katoh T, Suga H. Promoting ribosomal incorporation of backbone-modifying nonproteinogenic amino acids into nascent peptides by ATP-binding cassette family-F proteins and EF-P. Nucleic Acids Res. 2025;53:gkaf446. 10.1093/nar/gkaf446. 40401556 PMC12096078

[42] Katoh T, Wohlgemuth I, Nagano M et al. Essential structural elements in tRNA^Pro^ for EF-P-mediated alleviation of translation stalling. Nat Commun. 2016;7:11657. 10.1038/ncomms11657. 27216360 PMC4890201

[43] Doerfel LK, Wohlgemuth I, Kothe C et al. EF-P is essential for rapid synthesis of proteins containing consecutive proline residues. Science. 2013;339:85–8. 10.1126/science.1229017. 23239624

[44] Ude S, Lassak J, Starosta AL et al. Translation elongation factor EF-P alleviates ribosome stalling at polyproline stretches. Science. 2013;339:82–5. 10.1126/science.1228985. 23239623

[45] Crowe-McAuliffe C, Graf M, Huter P et al. Structural basis for antibiotic resistance mediated by the *Bacillus subtilis* ABCF ATPase VmlR. Proc Natl Acad Sci USA. 2018;115:8978–83. 10.1073/pnas.1808535115. 30126986 PMC6130385

[46] Crowe-McAuliffe C, Murina V, Turnbull KJ et al. Structural basis for PoxtA-mediated resistance to phenicol and oxazolidinone antibiotics. Nat Commun. 2022;13:1860. 10.1038/s41467-022-29274-9. 35387982 PMC8987054

[47] Crowe-McAuliffe C, Murina V, Turnbull KJ et al. Structural basis of ABCF-mediated resistance to pleuromutilin, lincosamide, and streptogramin A antibiotics in Gram-positive pathogens. Nat Commun. 2021;12:3577. 10.1038/s41467-021-23753-1. 34117249 PMC8196190

[48] Obana N, Takada H, Crowe-McAuliffe C et al. Genome-encoded ABCF factors implicated in intrinsic antibiotic resistance in Gram-positive bacteria: vmlR2, Ard1 and CplR. Nucleic Acids Res. 2023;51:4536–54. 10.1093/nar/gkad193. 36951104 PMC10201436

[49] Takada H, Fujiwara K, Atkinson GC et al. Resolution of ribosomal stalling by EF-P and ABCF ATPases YfmR and YkpA/YbiT. Nucleic Acids Res. 2024;52:9854–66. 10.1093/nar/gkae556. 38943426 PMC11381351

[50] Murina V, Kasari M, Hauryliuk V et al. Antibiotic resistance ABCF proteins reset the peptidyl transferase centre of the ribosome to counter translational arrest. Nucleic Acids Res. 2018;46:3753–63. 10.1093/nar/gky050. 29415157 PMC5909423

[51] Ignatov D, Shanmuganathan V, Ahmed-Begrich R et al. RNA-binding protein YebC enhances translation of proline-rich amino acid stretches in bacteria. Nat Commun. 2025;16:6262. 10.1038/s41467-025-60687-4. 40624002 PMC12234827

[52] Hong HR, Prince CR, Wu L et al. YebC2 resolves ribosome stalling and increases fitness of cells lacking EF-P and the ABCF ATPase YfmR. PLoS Genet. 2025;21:e1011633. 10.1371/journal.pgen.1011633. 40215226 PMC11990639

[53] Brischigliaro M, Kruger A, Moran JC et al. The human mitochondrial translation factor TACO1 alleviates mitoribosome stalling at polyproline stretches. Nucleic Acids Res. 2024;52:9710–26. 10.1093/nar/gkae645. 39036954 PMC11381339

[54] Katoh T, Takada H, Sigal M et al. The Alteromonas macleodii ribosome enables consecutive incorporation of bulky D-amino acids into peptides. Nucleic Acids Res. 2026;54:gkag341. 10.1093/nar/gkag341. 42011783 PMC13096806

[55] Charlton A, Zachariou M. Immobilized metal ion affinity chromatography of histidine-tagged fusion proteins. Methods Mol Biol. 2008;421:137–49. 18826053 10.1007/978-1-59745-582-4_10

[56] Block H, Maertens B, Spriestersbach A et al. Immobilized-metal affinity chromatography (IMAC): a review. Methods Enzymol. 2009;463:439–73. 19892187 10.1016/S0076-6879(09)63027-5

[57] Katoh T, Suga H. Reprogramming the genetic code with flexizymes. Nat Rev Chem. 2024;[8:879–92., 10.1038/s41570-024-00656-5 CrossRef] 39433956

[58] Katoh T, Suga H. Drop-off-reinitiation at the amino termini of nascent peptides and its regulation by IF3, EF-G, and RRF. RNA. 2023;29:663–74. 10.1261/rna.079447.122. 36754577 PMC10158994

[59] Tajima K, Katoh T, Suga H. Drop-off-reinitiation triggered by EF-G-driven mistranslocation and its alleviation by EF-P. Nucleic Acids Res. 2022;50:2736–53. 10.1093/nar/gkac068. 35188576 PMC8934632

[60] Katoh T, Suga H. Translation initiation with exotic amino acids using EF-P-responsive artificial initiator tRNA. Nucleic Acids Res. 2023;51:8169–80. 10.1093/nar/gkad496. 37334856 PMC10450175

[61] Goto Y, Ohta A, Sako Y et al. Reprogramming the translation initiation for the synthesis of physiologically stable cyclic peptides. ACS Chem Biol. 2008;3:120–9. 10.1021/cb700233t. 18215017

[62] Wang S, Brischigliaro M, Zhang Y et al. Structural basis of TACO1-mediated efficient mitochondrial translation. Nat Commun. 2026;17:2521. 41663403 10.1038/s41467-026-69156-y PMC12996406

[63] Huter P, Arenz S, Bock LV et al. Structural basis for polyproline-mediated ribosome stalling and rescue by the translation elongation factor EF-P. Mol Cell. 2017;68:515–527. 10.1016/j.molcel.2017.10.014. 29100052

[64] Hong HR, Prince CR, Tetreault DD et al. YfmR is a translation factor that prevents ribosome stalling and cell death in the absence of EF-P. Proc Natl Acad Sci USA. 2024;121:e2314437121. 10.1073/pnas.2314437121. 38349882 PMC10895253

[65] Chadani Y, Yamanouchi S, Uemura E et al. The ABCF proteins in *Escherichia coli* individually cope with ‘hard-to-translate’ nascent peptide sequences. Nucleic Acids Res. 2024;52:5825–40. 10.1093/nar/gkae309. 38661232 PMC11162784

[66] Watson ZL, Knudson IJ, Ward FR et al. Atomistic simulations of the *Escherichia coli* ribosome provide selection criteria for translationally active substrates. Nat Chem. 2023;15:913–21. 10.1038/s41557-023-01226-w. 37308707 PMC10322701

[67] Furukawa A, Schwochert J, Pye CR et al. Drug-like properties in macrocycles above MW 1000: backbone rigidity versus side-chain lipophilicity. Angew Chem Int Ed. 2020;59:21571–7. 10.1002/anie.202004550. PMC7719619 32789999

