# Supplementary material for: Enhancing ribosomal translation of backbone-altering nonproteinogenic amino acids via YebC and YeeN
Source: Nucleic Acids Res. 2026 Jun 22;54(12):gkag617. doi: 10.1093/nar/gkag617 (PMC13284719; doi:10.1093/nar/gkag617)
Supplement: gkag617_Supplemental_Files [file gkag617_supplemental_files.zip › 260529SI.pdf]

Supplementary Information for:

Enhancing ribosomal translation of backbone-altering nonproteinogenic amino acids via YebC and YeeN

Takayuki Katoh[1]* and Hiraku Takada[2]

[1]Department of Chemistry, Graduate School of Science, The University of Tokyo, Bunkyo-ku, Tokyo, Japan

[2]Department of Biotechnology, Faculty of Engineering, Toyama Prefectural University, Imizu-shi, Toyama, Japan

*To whom correspondence should be addressed. E-mail: katoh@chem.s.u-tokyo.ac.jp

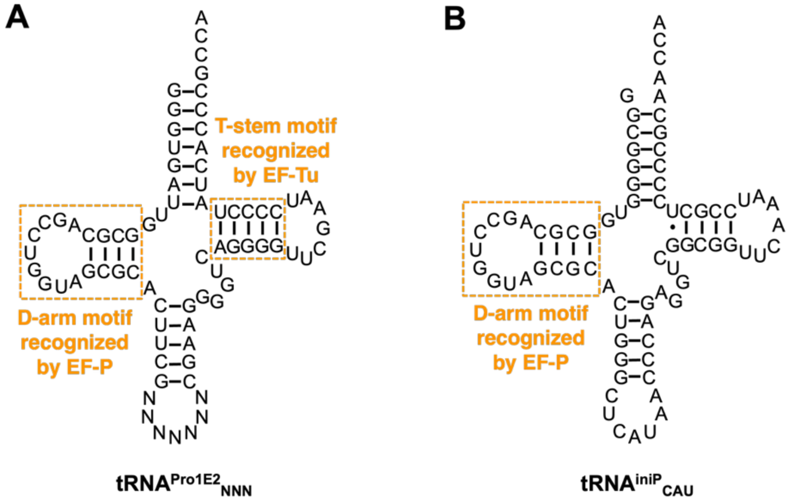

Supplementary Figure S1. Secondary structures of the engineered tRNAs used in this study. (**A**) $\text{tRNA}^{\text{Pro1E2}}$ used for BAA incorporation during translation elongation. The anticodon loop sequence was altered to recognize cognate codons. See Supplementary Table S1 for the specific anticodon loop sequences. (**B**) $\text{tRNA}^{\text{iniP}}$ used for ^{ClAc}L-Phe incorporation during translation initiation.

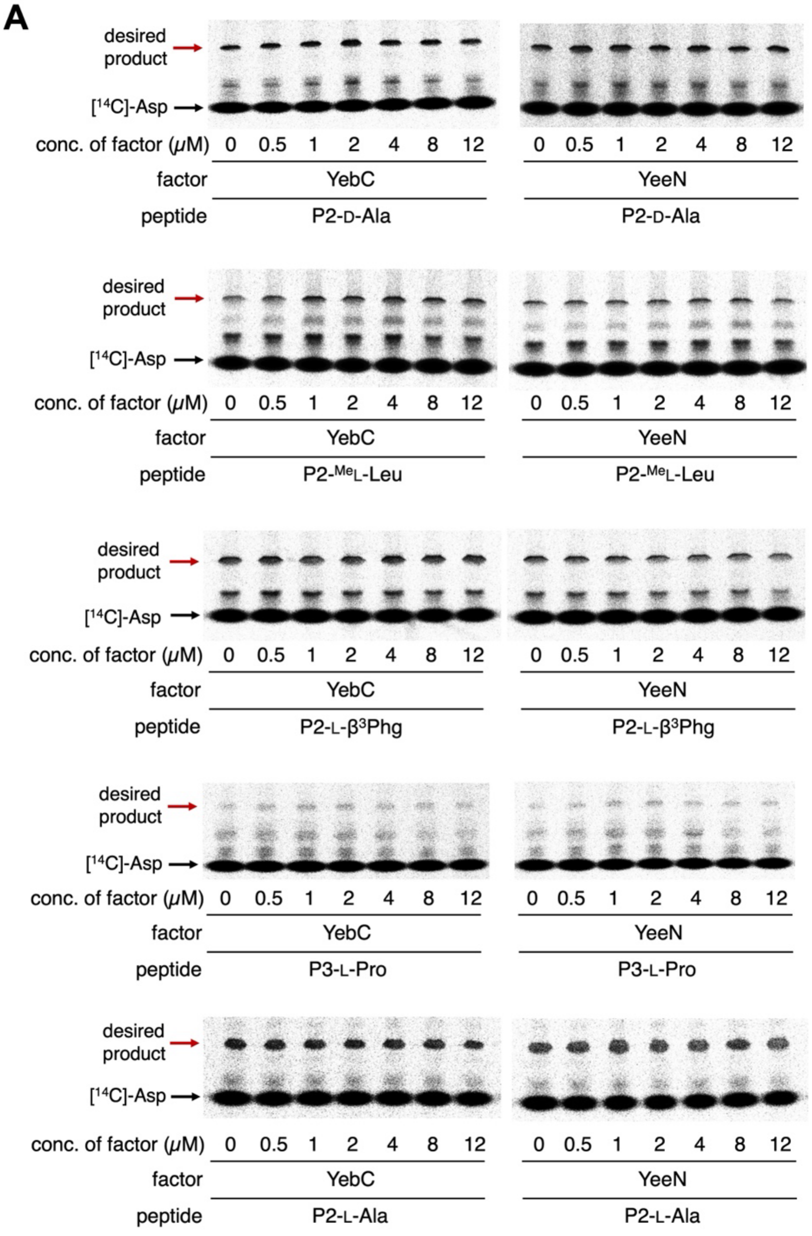

Supplementary Figure S2. Tricine SDS-PAGE analysis of translated peptides. (**A**) Titration of YebC or YeeN concentrations in the translation of P2 or P3. See Figure 2B−F for the quantification of the resulting peptides. (**B**) Incorporation of various BAAs into P2 using $tRNA^{Pro1E2}_{CGG}$ in the presence or absence of 2 μM YebC or YeeN. See Figure 3A,B for the quantification. **(C)** Combinations of YebC, YeeN, Uup, and EF-P in ^{Me}L-Leu incorporation into P2. The concentrations of these proteins were 2 μM, 2 μM, 1 μM, and 5 μM, respectively. See Figure 4B,C for the quantification. (**D**) ^{Me}L-Ala incorporation into P1'–6' and P2"–6" in the presence or absence of 2 μM YeeN. See Figure 5B for the quantification. (**E**) Translation of model macrocyclic peptides P7A and P7B in the presence or absence of 2 μM YebC, 2 μM YeeN, and/or 1 μM Uup. See Figure 7D for the quantification. (**F**) Titration of *A. macleodii* YebC or YeeN concentrations in the translation of P2-Ac$_4$c using the *A. macleodii* ribosome. See Figure 8A for the quantification. (**G**) Incorporation of various BAAs into P2 or P1 using the *A. macleodii* ribosome in the presence or absence of 2 μM *A. macleodii* YebC and/or YeeN. See also Figure 8B for the quantification.

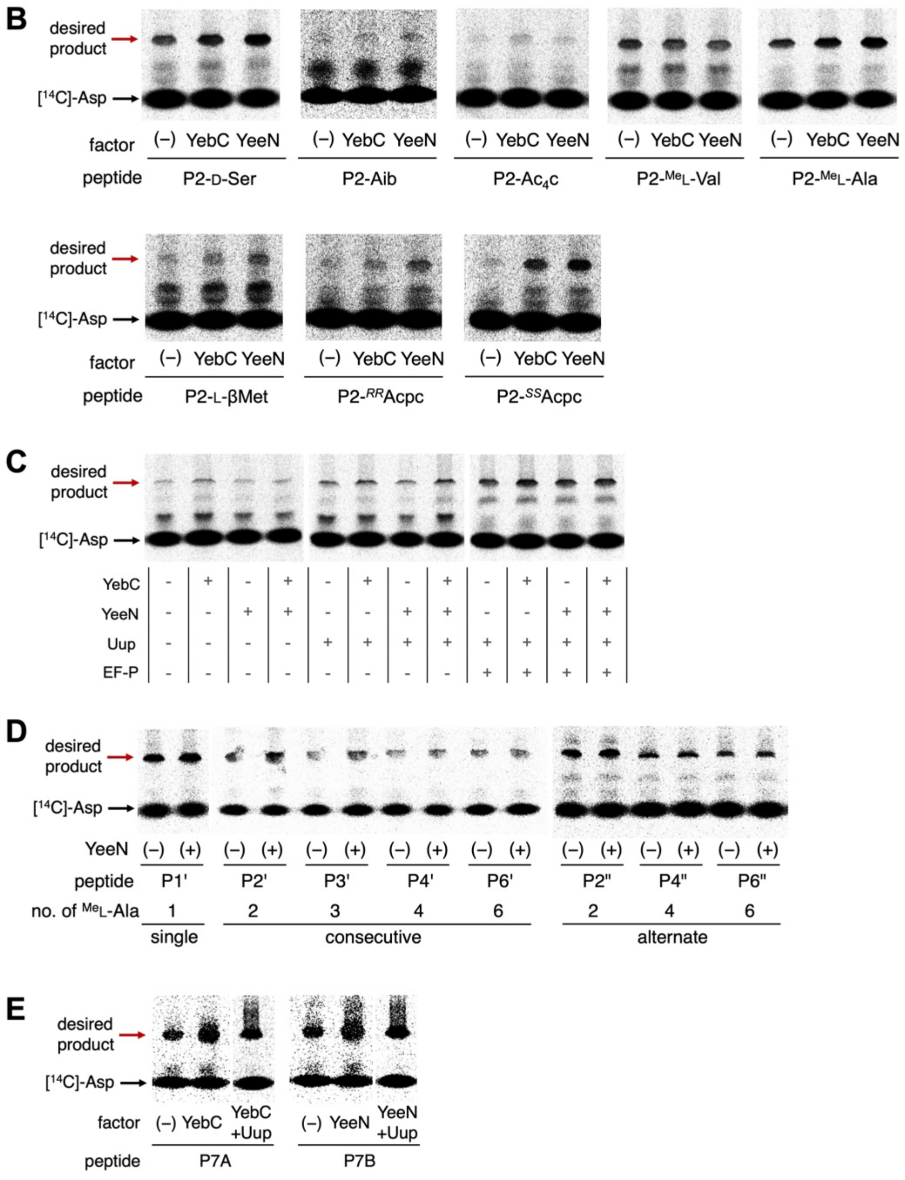

Supplementary Figure S2 continued.

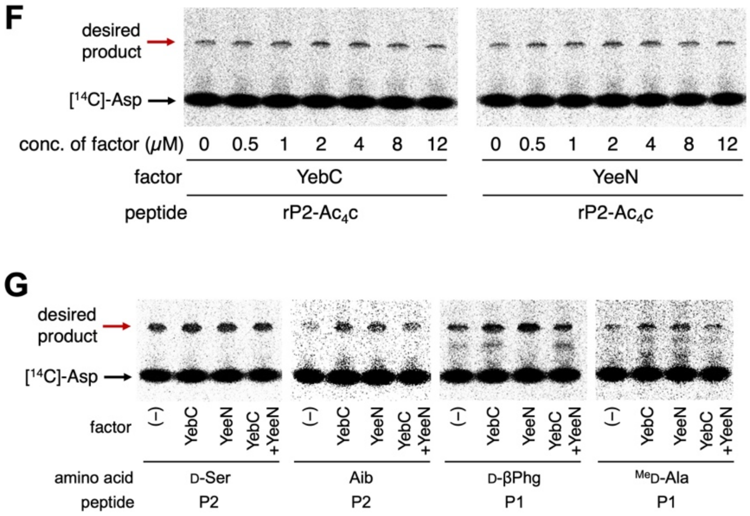

Supplementary Figure S2 continued.

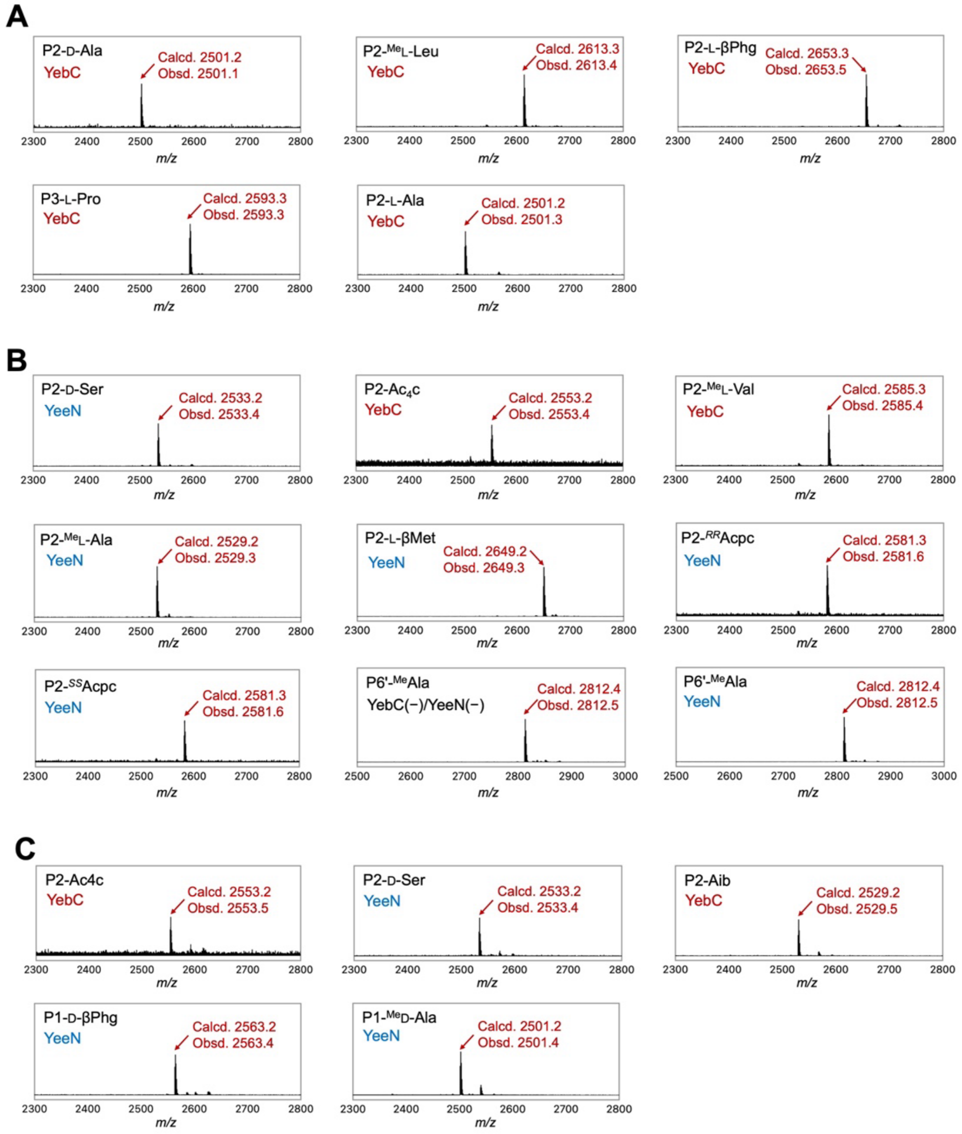

Supplementary Figure S3. MALDI-TOF MS analysis of model peptides. (**A, B**) Peptides translated using the *E. coli* ribosome and *E. coli* YebC or YeeN. (**C**) Peptides translated using the *A. macleodii* ribosome and *A. macleodii* YebC or YeeN. "Calcd." and "Obsd." indicate the calculated and observed $[M+H]^+$ values of the desired peptides, respectively.
